# Octa­butylbis[μ_2_-4-(diethyl­amino)­benzoato-κ^2^ *O*:*O*′]bis­[4-(diethyl­amino)­benzoato-κ*O*]di-μ_3_-oxido-tetra­tin(IV)

**DOI:** 10.1107/S1600536811025360

**Published:** 2011-07-02

**Authors:** Yip-Foo Win, Chen-Shang Choong, Sie-Tiong Ha, Chin Sing Yeap, Hoong-Kun Fun

**Affiliations:** a Department of Chemical Science, Faculty of Science, Universiti Tunku Abdul Rahman, Perak Campus, Jalan Universiti, Bandar Barat, 31900 Kampar, Perak, Malaysia; b X-ray Crystallography Unit, School of Physics, Universiti Sains Malaysia, 11800 USM, Penang, Malaysia

## Abstract

The asymmetric unit of the title complex, [Sn_4_(C_4_H_9_)_8_(C_11_H_14_NO_2_)_4_O_2_], consists of two crystallographically independent half-mol­ecules. The other halves are generated by crystallographic inversion centers. In each tetra­nuclear mol­ecule, both of the two independent Sn atoms are five-coordinated, with distorted trigonal–bipyramidal SnC_2_O_3_ geometries. One Sn atom is coordinated by two butyl groups, one O atom of the benzoate anion and two bridging O atoms, whereas the other Sn atom is coordinated by two butyl groups, two O atoms of the benzoate anions and a bridging O atom. All the butyl groups are equatorial with respect to the SnO_3_ trigonal plane. Weak intra­molecular C—H⋯O hydrogen bonds stabilize the mol­ecular structures. In one mol­ecule, two of the butyl groups and the bridging benzoate anion are each disordered over two positions.

## Related literature

For general background to and applications of the title complex, see: Khoo & Hazell (1999 ▶); Parvez *et al.* (2004 ▶); Li *et al.* (2006 ▶). For closely related structures, see: Win *et al.* (2008 ▶, 2010*a* ▶,*b* ▶). For the stability of the temperature controller used in the data collection, see: Cosier & Glazer (1986 ▶).
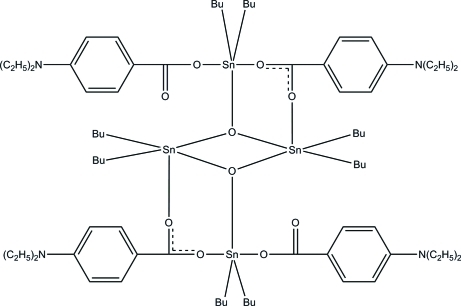


## Experimental

### 

#### Crystal data

[Sn_4_(C_4_H_9_)_8_(C_11_H_14_NO_2_)_4_O_2_]
*M* *_r_* = 1732.58
Triclinic, 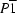

*a* = 11.7394 (1) Å
*b* = 14.4449 (1) Å
*c* = 24.3248 (3) Å
α = 102.374 (1)°
β = 90.100 (1)°
γ = 92.164 (1)°
*V* = 4025.96 (7) Å^3^
*Z* = 2
Mo *K*α radiation
μ = 1.28 mm^−1^
*T* = 100 K
0.30 × 0.22 × 0.10 mm

#### Data collection

Bruker SMART APEXII CCD area-detector diffractometer
Absorption correction: multi-scan (*SADABS*; Bruker, 2009 ▶) *T*_min_ = 0.697, *T*_max_ = 0.883
52692 measured reflections
15818 independent reflections
13267 reflections with *I* > 2σ(*I*)
*R*_int_ = 0.034

#### Refinement

*R*[*F* ^2^ > 2σ(*F* ^2^)] = 0.033
*wR*(*F* ^2^) = 0.073
*S* = 1.02
15818 reflections
910 parameters
96 restraints
H-atom parameters constrained
Δρ_max_ = 2.27 e Å^−3^
Δρ_min_ = −1.97 e Å^−3^

### 

Data collection: *APEX2* (Bruker, 2009 ▶); cell refinement: *SAINT* (Bruker, 2009 ▶); data reduction: *SAINT*; program(s) used to solve structure: *SHELXTL* (Sheldrick, 2008 ▶); program(s) used to refine structure: *SHELXTL*; molecular graphics: *SHELXTL*; software used to prepare material for publication: *SHELXTL* and *PLATON* (Spek, 2009 ▶).

## Supplementary Material

Crystal structure: contains datablock(s) global, I. DOI: 10.1107/S1600536811025360/wm2503sup1.cif

Structure factors: contains datablock(s) I. DOI: 10.1107/S1600536811025360/wm2503Isup2.hkl

Additional supplementary materials:  crystallographic information; 3D view; checkCIF report

## Figures and Tables

**Table 1:** Hydrogen-bond geometry (Å, °)

*D*—H⋯*A*	*D*—H	H⋯*A*	*D*⋯*A*	*D*—H⋯*A*
C28*A*—H28*A*⋯O3*A*	0.99	2.56	3.140 (4)	117
C28*B*—H28*D*⋯O3*B*	0.99	2.43	3.065 (8)	121
C32*A*—H32*B*⋯O2*A*	0.99	2.47	3.252 (5)	136

**Fig. 1.**
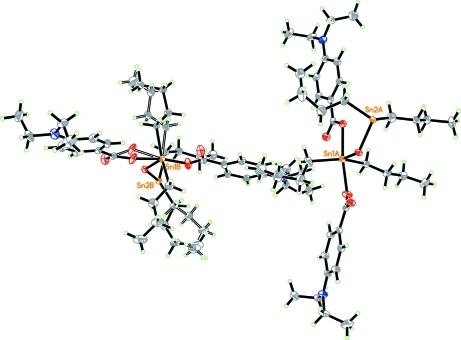
The asymmetric unit of the title compound, with 50% probability ellipsoids for non-H atoms. All disorder components are shown.

**Fig. 2.**
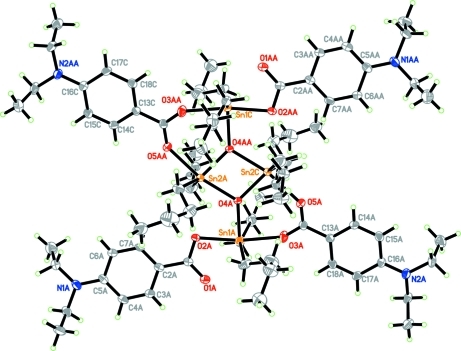
One of the two whole molecules of title compound, with 50% probability ellipsoids for non-H atoms. [Symmetry code A) -x+1, -y+2, -z.]

## supplementary crystallographic information

### Comment

In general, there are many well-documented structures on complexes isolated from the 1:1 molar ratio reaction between diorganotin(IV) with the respective organic acids. Commonly, the resulting dimeric structure is known as the organodistannoxane dimer (Khoo & Hazell, 1999; Parvez *et al.*, 2004; Li *et al.*, 2006). The core geometry of the organodistannoxane dimer complexes consists of a centrosymmetric planar Sn_2_O_2_ group bonded to the exo- and endocyclic tin(IV) atom moiety *via* the bridging oxygen atoms so that the oxygen atoms are tri-coordinated (Win *et al.*, 2008; 2010*a*,*b*). An exception is when 4-(diethylamino)benzoic acid is utilized in the synthesis to obtain the title complex.

The asymmetric unit of the title complex consists of two crystallographically independent half-molecules, *A* and *B* (Fig. 1). The other halves are generated by crystallographic inversion centers (Fig. 2). Similar to previous structures (Win *et al.*, 2008; 2010*a*,*b*), both Sn atoms in each molecule are five-coordinated in distorted trigonal-bipyramidal geometries but their coordination environments are different. The Sn1 atom is coordinated by two butyl groups in equatorial positions, an O atom of the monodentate benzoate anion, an O atom of the bridging benzoate anion and one oxido-bridged O atom whereas the Sn2 atom is coordinated by two butyl groups in equatorial positions, an O atom of the bridging benzoate anion and two oxido-bridged O atoms. Weak intramolecular C28A—H28A···O3A, C28B—H28D···O3B and C32A—H32B···O2A hydrogen bonds (Table 1) stabilize the molecular structures. There is no significant intermolecular hydrogen bond observed.

### Experimental

The title complex was obtained by heating under reflux a 1:1 molar mixture of dibutyltin(IV) oxide (0.50 g, 2 mmole) and 4-(diethylamino)benzoic acid (0.39 g, 2 mmole) in ethanol (50 ml) for two hours. A clear transparent solution was isolated by filtration and kept in a bottle. After five days, colourless crystals (2.96 g, 85.3% yield) were collected. Melting point: 208.3–209.9 °C. Analysis for C_76_H_128_N_4_O_10_Sn_4_: C, 52.78; H, 7.07; N, 3.19; Sn, 27.37%. Calculated for C_76_H_128_N_4_O_10_Sn_4_: C, 52.68; H, 7.45; N, 3.23; Sn, 27.41%. FTIR as KBr disc (cm^-1^): ν(C—H) aromatic 3082, 3052; ν(C—H) saturated 2957, 2925, 2869; ν(COO)_as_ 1604, 1576; ν(COO)_s_ 1350, 1394; ν(C—N) 1269, ν(Sn—O—Sn) 634, ν(Sn—C) 548, ν(Sn—O) 468. ^1^H-NMR (p.p.m.) (CDCl_3_): δ: benzene protons 6.66 (d, 6.9 Hz, 8H); 7.91 (d, 7.3 Hz, 8H); butyl, CH_3_ 0.88 (t, 7.3 Hz, 24H); CH_2_ 1.37–1.41 (m, 16H); CH_2_ 1.57–1.74 (m, 32H); N-(CH_2_CH_3_)_2_ 1.23 (t, 6.5 Hz, 24H); 3.44 (q, 6.8 Hz, 16H). ^13^C-NMR (p.p.m.) (CDCl_3_): δ: benzene carbons 110.43, 120.03, 132.36, 150.78; butyl 14.09, 26.83, 27.13, 27.27, 27.42, 27.90, 28.45; N-(CH_2_CH_3_)_2_ 12.95, 44.87; COO 173.44. ^119^Sn-NMR (p.p.m.) (CDCl_3_): δ: -171.65, -221.43.

### Refinement

All hydrogen atoms were positioned geomatrically [C–H = 0.95–0.99 Å] and refined using a riding model, with *U*_iso_(H) = 1.2 or 1.5 *U*_eq_(C). A rotating-group model were applied for methyl groups. The same *U*_ij_ parameters were used for atom pairs C34A/C35A, C36B/C36Y, C37B C37Y, C38B/C38Y and O3B/O3Y. The C23B–C24B–C25B–C26B and C23Y–C24Y–C25Y–C26Y butyl chains were subjected to rigid bond and similarity restraints. For molecule *B*, two of the butyl groups are disordered over two positions with a refined site-occupancies ratio of 0.665 (7):0.335 (7) and 0.780 (5):0.220 (5), respectively. One of the O atoms of the bridging benzoate anion is disorderd over two positions with refined site-occupancy ratio of 0.780 (15):0.220 (15). The maximum and minimum residual electron density peaks of 2.27 and -1.97 e° A^-3^ are located 0.05 Å and 0.09 Å from atoms C35A and C34A, respectively. Fifteen outliners, (0 0 2), (-11 12 2), (-13 12 2), (-12 11 2), (7 16 4), (-11 13 2), (-11 11 2), (2 19 4), (0 -1 1), (1 6 29), (-14 11 2), (0 1 1), (7 17 4), (-9 1 1) and (14 10 0) were omitted.

### Crystal data

**Table:**

[Sn_4_(C_4_H_9_)_8_(C_11_H_14_NO_2_)_4_O_2_]	*Z* = 2
*M**_r_* = 1732.58	*F*(000) = 1784
Triclinic, *P*1	*D*_x_ = 1.429 Mg m^−^^3^
Hall symbol: -P 1	Mo *K*α radiation, λ = 0.71073 Å
*a* = 11.7394 (1) Å	Cell parameters from 9838 reflections
*b* = 14.4449 (1) Å	θ = 2.2–30.1°
*c* = 24.3248 (3) Å	µ = 1.28 mm^−^^1^
α = 102.374 (1)°	*T* = 100 K
β = 90.100 (1)°	Block, colourless
γ = 92.164 (1)°	0.30 × 0.22 × 0.10 mm
*V* = 4025.96 (7) Å^3^	

### Data collection

**Table:**

Bruker SMART APEXII CCD area-detector diffractometer	15818 independent reflections
Radiation source: fine-focus sealed tube	13267 reflections with *I* > 2σ(*I*)
graphite	*R*_int_ = 0.034
φ and ω scans	θ_max_ = 26.0°, θ_min_ = 0.9°
Absorption correction: multi-scan (*SADABS*; Bruker, 2009)	*h* = −14→13
*T*_min_ = 0.697, *T*_max_ = 0.883	*k* = −17→17
52692 measured reflections	*l* = −30→28

### Refinement

**Table:**

Refinement on *F*^2^	Primary atom site location: structure-invariant direct methods
Least-squares matrix: full	Secondary atom site location: difference Fourier map
*R*[*F*^2^ > 2σ(*F*^2^)] = 0.033	Hydrogen site location: inferred from neighbouring sites
*wR*(*F*^2^) = 0.073	H-atom parameters constrained
*S* = 1.02	*w* = 1/[σ^2^(*F*_o_^2^) + (0.0261*P*)^2^ + 6.8533*P*] where *P* = (*F*_o_^2^ + 2*F*_c_^2^)/3
15818 reflections	(Δ/σ)_max_ = 0.002
910 parameters	Δρ_max_ = 2.27 e Å^−^^3^
96 restraints	Δρ_min_ = −1.97 e Å^−^^3^

### Special details

**Table:**

Experimental. The crystal was placed in the cold stream of an Oxford Cryosystems Cobra open-flow nitrogen cryostat (Cosier & Glazer, 1986) operating at 100.0 (1) K.
Geometry. All e.s.d.'s (except the e.s.d. in the dihedral angle between two l.s. planes) are estimated using the full covariance matrix. The cell e.s.d.'s are taken into account individually in the estimation of e.s.d.'s in distances, angles and torsion angles; correlations between e.s.d.'s in cell parameters are only used when they are defined by crystal symmetry. An approximate (isotropic) treatment of cell e.s.d.'s is used for estimating e.s.d.'s involving l.s. planes.
Refinement. Refinement of *F*^2^ against ALL reflections. The weighted *R*-factor *wR* and goodness of fit *S* are based on *F*^2^, conventional *R*-factors *R* are based on *F*, with *F* set to zero for negative *F*^2^. The threshold expression of *F*^2^ > σ(*F*^2^) is used only for calculating *R*-factors(gt) *etc*. and is not relevant to the choice of reflections for refinement. *R*-factors based on *F*^2^ are statistically about twice as large as those based on *F*, and *R*- factors based on ALL data will be even larger.

### Fractional atomic coordinates and isotropic or equivalent isotropic displacement parameters (Å^2^)

**Table:**

	*x*	*y*	*z*	*U*_iso_*/*U*_eq_	Occ. (<1)
Sn1A	0.478986 (18)	0.809151 (15)	0.055867 (9)	0.01723 (6)	
Sn2A	0.391885 (18)	1.045239 (15)	0.040096 (9)	0.01598 (6)	
O1A	0.35629 (19)	0.76271 (16)	0.13734 (10)	0.0236 (5)	
O2A	0.36192 (19)	0.89993 (15)	0.11042 (9)	0.0193 (5)	
O3A	0.59208 (19)	0.72863 (16)	−0.00960 (10)	0.0261 (5)	
O4A	0.50617 (18)	0.92753 (14)	0.02312 (9)	0.0178 (5)	
O5A	0.71510 (19)	0.82851 (15)	−0.03965 (10)	0.0231 (5)	
N1A	−0.0066 (2)	0.9869 (2)	0.30775 (12)	0.0251 (7)	
N2A	0.9622 (2)	0.43136 (19)	−0.11082 (12)	0.0241 (6)	
C1A	0.3217 (3)	0.8447 (2)	0.14233 (13)	0.0188 (7)	
C2A	0.2364 (3)	0.8830 (2)	0.18500 (13)	0.0193 (7)	
C3A	0.1989 (3)	0.8300 (2)	0.22419 (14)	0.0219 (7)	
H3AA	0.2286	0.7693	0.2226	0.026*	
C4A	0.1208 (3)	0.8639 (2)	0.26451 (14)	0.0235 (8)	
H4AA	0.0982	0.8264	0.2906	0.028*	
C5A	0.0732 (3)	0.9533 (2)	0.26819 (14)	0.0221 (7)	
C6A	0.1106 (3)	1.0064 (2)	0.22859 (14)	0.0247 (8)	
H6AA	0.0805	1.0668	0.2295	0.030*	
C7A	0.1903 (3)	0.9715 (2)	0.18873 (14)	0.0216 (7)	
H7AA	0.2145	1.0091	0.1630	0.026*	
C8A	−0.0500 (3)	0.9322 (2)	0.34796 (15)	0.0288 (8)	
H8AA	−0.0511	0.8638	0.3300	0.035*	
H8AB	−0.1293	0.9495	0.3577	0.035*	
C9A	0.0213 (4)	0.9490 (3)	0.40155 (15)	0.0356 (9)	
H9AA	−0.0108	0.9106	0.4269	0.053*	
H9AB	0.0209	1.0163	0.4201	0.053*	
H9AC	0.0998	0.9311	0.3923	0.053*	
C10A	−0.0506 (3)	1.0821 (3)	0.31406 (15)	0.0295 (8)	
H10A	0.0109	1.1255	0.3055	0.035*	
H10B	−0.0730	1.1053	0.3537	0.035*	
C11A	−0.1524 (4)	1.0852 (3)	0.27605 (18)	0.0439 (11)	
H11A	−0.1801	1.1498	0.2832	0.066*	
H11B	−0.2132	1.0415	0.2838	0.066*	
H11C	−0.1296	1.0663	0.2366	0.066*	
C12A	0.6853 (3)	0.7465 (2)	−0.03204 (13)	0.0192 (7)	
C13A	0.7614 (3)	0.6667 (2)	−0.05067 (13)	0.0188 (7)	
C14A	0.8547 (3)	0.6736 (2)	−0.08525 (14)	0.0209 (7)	
H14A	0.8730	0.7324	−0.0953	0.025*	
C15A	0.9207 (3)	0.5970 (2)	−0.10509 (14)	0.0230 (7)	
H15A	0.9827	0.6037	−0.1292	0.028*	
C16A	0.8983 (3)	0.5088 (2)	−0.09036 (14)	0.0199 (7)	
C17A	0.8071 (3)	0.5037 (2)	−0.05348 (14)	0.0223 (7)	
H17A	0.7914	0.4463	−0.0413	0.027*	
C18A	0.7401 (3)	0.5802 (2)	−0.03471 (14)	0.0210 (7)	
H18A	0.6782	0.5741	−0.0105	0.025*	
C19A	1.0610 (3)	0.4370 (3)	−0.14608 (16)	0.0307 (9)	
H19A	1.1155	0.3891	−0.1404	0.037*	
H19B	1.0994	0.5004	−0.1339	0.037*	
C20A	1.0318 (4)	0.4209 (3)	−0.20813 (17)	0.0433 (11)	
H20A	1.1018	0.4244	−0.2296	0.065*	
H20B	0.9806	0.4698	−0.2144	0.065*	
H20C	0.9940	0.3582	−0.2206	0.065*	
C21A	0.9296 (3)	0.3371 (2)	−0.10203 (15)	0.0249 (8)	
H21A	0.9558	0.2889	−0.1343	0.030*	
H21B	0.8454	0.3307	−0.1009	0.030*	
C22A	0.9790 (3)	0.3175 (3)	−0.04801 (16)	0.0334 (9)	
H22A	0.9555	0.2531	−0.0446	0.050*	
H22B	0.9510	0.3634	−0.0157	0.050*	
H22C	1.0624	0.3232	−0.0489	0.050*	
C23A	0.6087 (3)	0.8180 (3)	0.11947 (17)	0.0338 (9)	
H23A	0.6018	0.8794	0.1464	0.041*	
H23E	0.5908	0.7675	0.1403	0.041*	
C24A	0.7303 (3)	0.8102 (3)	0.10251 (17)	0.0418 (10)	
H24A	0.7415	0.8404	0.0699	0.050*	
H24B	0.7461	0.7422	0.0897	0.050*	
C25A	0.8194 (4)	0.8555 (3)	0.14889 (18)	0.0451 (11)	
H25A	0.8969	0.8426	0.1340	0.054*	
H25B	0.8116	0.9251	0.1579	0.054*	
C26A	0.8066 (4)	0.8195 (3)	0.20097 (18)	0.0475 (11)	
H26A	0.8656	0.8496	0.2282	0.071*	
H26B	0.8146	0.7507	0.1924	0.071*	
H26C	0.7312	0.8345	0.2169	0.071*	
C27A	0.3490 (3)	0.7202 (2)	0.00821 (14)	0.0218 (7)	
H27A	0.3626	0.6537	0.0101	0.026*	
H27B	0.2748	0.7368	0.0262	0.026*	
C28A	0.3401 (3)	0.7258 (2)	−0.05356 (14)	0.0232 (8)	
H28A	0.4132	0.7070	−0.0721	0.028*	
H28B	0.3285	0.7925	−0.0557	0.028*	
C29A	0.2436 (3)	0.6633 (3)	−0.08579 (15)	0.0299 (8)	
H29A	0.2590	0.5960	−0.0870	0.036*	
H29B	0.1714	0.6778	−0.0653	0.036*	
C30A	0.2294 (3)	0.6769 (3)	−0.14536 (16)	0.0375 (10)	
H30A	0.1669	0.6349	−0.1641	0.056*	
H30B	0.3001	0.6616	−0.1661	0.056*	
H30C	0.2119	0.7430	−0.1445	0.056*	
C31A	0.4474 (3)	1.1223 (2)	0.12122 (15)	0.0288 (8)	
H31A	0.4773	1.1853	0.1168	0.035*	
H31C	0.3790	1.1333	0.1452	0.035*	
C32A	0.5351 (4)	1.0824 (3)	0.15328 (18)	0.0395 (10)	
H32A	0.6064	1.0754	0.1314	0.047*	
H32B	0.5082	1.0184	0.1572	0.047*	
C33A	0.5614 (4)	1.1447 (4)	0.2129 (2)	0.0584 (13)	
H33A	0.6216	1.1139	0.2303	0.070*	
H33B	0.5940	1.2065	0.2080	0.070*	
C34A	0.4690 (4)	1.1634 (3)	0.25254 (17)	0.0431 (7)	
H34A	0.4991	1.1978	0.2891	0.065*	
H34B	0.4323	1.1032	0.2566	0.065*	
H34C	0.4130	1.2018	0.2387	0.065*	
C35A	0.2366 (4)	0.9655 (3)	0.01562 (17)	0.0431 (7)	
H35A	0.2506	0.8979	0.0143	0.052*	
H35D	0.1800	0.9857	0.0455	0.052*	
C36A	0.1832 (3)	0.9725 (2)	−0.04028 (14)	0.0225 (7)	
H36A	0.2402	0.9562	−0.0704	0.027*	
H36B	0.1621	1.0388	−0.0384	0.027*	
C37A	0.0770 (3)	0.9070 (2)	−0.05560 (15)	0.0250 (8)	
H37A	0.0983	0.8405	−0.0582	0.030*	
H37B	0.0204	0.9225	−0.0252	0.030*	
C38A	0.0227 (3)	0.9156 (3)	−0.11100 (16)	0.0361 (9)	
H38A	−0.0484	0.8770	−0.1172	0.054*	
H38B	0.0752	0.8934	−0.1419	0.054*	
H38C	0.0064	0.9821	−0.1096	0.054*	
Sn1B	0.959478 (19)	0.692225 (15)	0.448378 (9)	0.01771 (6)	
Sn2B	0.898606 (18)	0.441889 (14)	0.456896 (9)	0.01604 (6)	
O1B	0.8255 (2)	0.73707 (17)	0.37546 (11)	0.0339 (6)	
O2B	0.8252 (2)	0.59941 (17)	0.40134 (11)	0.0306 (6)	
O3B	1.1160 (5)	0.7740 (3)	0.4914 (3)	0.0286 (13)	0.780 (15)
O3Y	1.0790 (18)	0.7887 (12)	0.5106 (8)	0.0286 (13)	0.220 (15)
O4B	0.98666 (18)	0.57986 (14)	0.48493 (9)	0.0177 (5)	
O5B	1.1587 (2)	0.70917 (17)	0.56343 (11)	0.0341 (6)	
N1B	0.4967 (3)	0.4932 (2)	0.18318 (12)	0.0284 (7)	
N2B	1.4711 (3)	1.0901 (2)	0.62086 (14)	0.0319 (7)	
C1B	0.7917 (3)	0.6525 (2)	0.36766 (15)	0.0265 (8)	
C2B	0.7168 (3)	0.6084 (2)	0.31938 (14)	0.0236 (8)	
C3B	0.6831 (3)	0.6624 (2)	0.28178 (15)	0.0252 (8)	
H3BA	0.7087	0.7270	0.2875	0.030*	
C4B	0.6136 (3)	0.6243 (2)	0.23650 (15)	0.0264 (8)	
H4BA	0.5938	0.6629	0.2111	0.032*	
C5B	0.5709 (3)	0.5296 (2)	0.22669 (14)	0.0236 (7)	
C6B	0.6086 (3)	0.4747 (2)	0.26424 (14)	0.0241 (8)	
H6BA	0.5840	0.4099	0.2586	0.029*	
C7B	0.6801 (3)	0.5132 (2)	0.30873 (14)	0.0235 (7)	
H7BA	0.7052	0.4741	0.3328	0.028*	
C8B	0.4540 (4)	0.5513 (3)	0.14565 (16)	0.0345 (9)	
H8BA	0.4498	0.6178	0.1667	0.041*	
H8BB	0.3760	0.5282	0.1328	0.041*	
C9B	0.5295 (4)	0.5482 (3)	0.09489 (17)	0.0440 (11)	
H9BA	0.4979	0.5874	0.0708	0.066*	
H9BB	0.5332	0.4826	0.0737	0.066*	
H9BC	0.6064	0.5727	0.1074	0.066*	
C10B	0.4482 (3)	0.3966 (2)	0.17364 (15)	0.0288 (8)	
H10C	0.5061	0.3545	0.1834	0.035*	
H10D	0.4292	0.3746	0.1332	0.035*	
C11B	0.3412 (3)	0.3883 (3)	0.20814 (17)	0.0398 (10)	
H11D	0.3091	0.3232	0.1981	0.060*	
H11E	0.2849	0.4321	0.2001	0.060*	
H11F	0.3610	0.4041	0.2483	0.060*	
C12B	1.1666 (3)	0.7756 (2)	0.53745 (14)	0.0206 (7)	
C13B	1.2455 (3)	0.8578 (2)	0.55953 (14)	0.0196 (7)	
C14B	1.3227 (3)	0.8564 (2)	0.60248 (15)	0.0266 (8)	
H14B	1.3240	0.8018	0.6184	0.032*	
C15B	1.3975 (3)	0.9315 (2)	0.62283 (16)	0.0292 (8)	
H15B	1.4495	0.9278	0.6522	0.035*	
C16B	1.3978 (3)	1.0142 (2)	0.60059 (15)	0.0253 (8)	
C17B	1.3190 (3)	1.0151 (2)	0.55708 (15)	0.0249 (8)	
H17B	1.3165	1.0696	0.5412	0.030*	
C18B	1.2457 (3)	0.9388 (2)	0.53698 (14)	0.0223 (7)	
H18B	1.1942	0.9413	0.5072	0.027*	
C19B	1.5547 (3)	1.0878 (3)	0.66552 (19)	0.0418 (11)	
H19C	1.6184	1.1337	0.6633	0.050*	
H19D	1.5862	1.0238	0.6591	0.050*	
C20B	1.5048 (4)	1.1113 (3)	0.72378 (19)	0.0518 (12)	
H20D	1.5633	1.1060	0.7516	0.078*	
H20E	1.4406	1.0670	0.7261	0.078*	
H20F	1.4780	1.1762	0.7314	0.078*	
C21B	1.4625 (3)	1.1801 (2)	0.60311 (16)	0.0299 (9)	
H21C	1.4887	1.2326	0.6343	0.036*	
H21D	1.3817	1.1897	0.5949	0.036*	
C22B	1.5328 (4)	1.1834 (3)	0.55145 (19)	0.0526 (13)	
H22D	1.5191	1.2422	0.5390	0.079*	
H22E	1.5110	1.1288	0.5213	0.079*	
H22F	1.6139	1.1811	0.5606	0.079*	
C23B	0.8759 (5)	0.7919 (4)	0.5115 (3)	0.0177 (13)	0.665 (7)
H23B	0.9146	0.7937	0.5479	0.021*	0.665 (7)
H23F	0.8879	0.8554	0.5028	0.021*	0.665 (7)
C24B	0.7498 (4)	0.7759 (4)	0.5194 (2)	0.0250 (14)	0.665 (7)
H24C	0.7361	0.7140	0.5300	0.030*	0.665 (7)
H24D	0.7093	0.7736	0.4834	0.030*	0.665 (7)
C25B	0.7010 (5)	0.8551 (4)	0.5653 (3)	0.0243 (14)	0.665 (7)
H25C	0.7224	0.9175	0.5569	0.029*	0.665 (7)
H25D	0.6167	0.8482	0.5645	0.029*	0.665 (7)
C26B	0.7441 (6)	0.8527 (5)	0.6244 (3)	0.0264 (16)	0.665 (7)
H26D	0.7067	0.9011	0.6522	0.040*	0.665 (7)
H26E	0.8268	0.8654	0.6266	0.040*	0.665 (7)
H26F	0.7265	0.7900	0.6323	0.040*	0.665 (7)
C23Y	0.8311 (12)	0.7742 (10)	0.4947 (5)	0.031 (3)	0.335 (7)
H23C	0.8408	0.8402	0.4894	0.037*	0.335 (7)
H23D	0.7559	0.7490	0.4785	0.037*	0.335 (7)
C24Y	0.8296 (9)	0.7767 (7)	0.5572 (4)	0.030 (3)	0.335 (7)
H24E	0.9059	0.7985	0.5733	0.036*	0.335 (7)
H24F	0.8145	0.7115	0.5628	0.036*	0.335 (7)
C25Y	0.7397 (11)	0.8419 (9)	0.5891 (7)	0.034 (3)	0.335 (7)
H25E	0.7575	0.9078	0.5853	0.041*	0.335 (7)
H25F	0.6640	0.8223	0.5716	0.041*	0.335 (7)
C26Y	0.7343 (17)	0.8397 (14)	0.6516 (7)	0.056 (5)	0.335 (7)
H26G	0.6729	0.8794	0.6693	0.084*	0.335 (7)
H26H	0.8071	0.8639	0.6699	0.084*	0.335 (7)
H26I	0.7194	0.7743	0.6557	0.084*	0.335 (7)
C27B	1.0703 (3)	0.6812 (3)	0.37794 (16)	0.0311 (9)	
H27C	1.0290	0.6462	0.3438	0.037*	
H27D	1.0899	0.7458	0.3723	0.037*	
C28B	1.1788 (3)	0.6321 (3)	0.38403 (17)	0.0344 (9)	
H28C	1.1596	0.5655	0.3860	0.041*	
H28D	1.2167	0.6634	0.4200	0.041*	
C29B	1.2633 (4)	0.6325 (3)	0.33551 (19)	0.0449 (11)	
H29C	1.2271	0.5996	0.2994	0.054*	
H29D	1.2828	0.6987	0.3330	0.054*	
C30B	1.3711 (4)	0.5831 (3)	0.3452 (2)	0.0544 (13)	
H30D	1.4249	0.5860	0.3148	0.082*	
H30E	1.3521	0.5167	0.3458	0.082*	
H30F	1.4059	0.6148	0.3813	0.082*	
C31B	0.7441 (3)	0.4866 (2)	0.49825 (15)	0.0246 (8)	
H31B	0.7584	0.4986	0.5393	0.030*	
H31D	0.7257	0.5480	0.4892	0.030*	
C32B	0.6392 (3)	0.4202 (3)	0.48489 (15)	0.0289 (8)	
H32C	0.5777	0.4443	0.5113	0.035*	
H32D	0.6580	0.3567	0.4906	0.035*	
C33B	0.5964 (3)	0.4111 (3)	0.42473 (16)	0.0316 (9)	
H33C	0.6553	0.3811	0.3985	0.038*	
H33D	0.5857	0.4754	0.4179	0.038*	
C34B	0.4843 (3)	0.3528 (3)	0.41177 (19)	0.0442 (11)	
H34D	0.4601	0.3518	0.3730	0.066*	
H34E	0.4256	0.3816	0.4379	0.066*	
H34F	0.4953	0.2878	0.4161	0.066*	
C35B	0.9637 (3)	0.4163 (3)	0.37337 (15)	0.0300 (8)	
H35B	0.9010	0.3880	0.3471	0.036*	0.780 (5)
H35C	0.9863	0.4782	0.3646	0.036*	0.780 (5)
H35E	1.0443	0.4082	0.3754	0.036*	0.220 (5)
H35F	0.9532	0.4720	0.3587	0.036*	0.220 (5)
C36B	1.0620 (4)	0.3539 (4)	0.3617 (2)	0.0334 (11)	0.780 (5)
H36C	1.0369	0.2890	0.3648	0.040*	0.780 (5)
H36D	1.1213	0.3768	0.3910	0.040*	0.780 (5)
C37B	1.1153 (5)	0.3492 (4)	0.3035 (2)	0.0378 (12)	0.780 (5)
H37C	1.1477	0.4128	0.3020	0.045*	0.780 (5)
H37D	1.1790	0.3052	0.2991	0.045*	0.780 (5)
C38B	1.0336 (10)	0.3171 (18)	0.2549 (6)	0.047 (2)	0.780 (5)
H38D	1.0752	0.3104	0.2194	0.070*	0.780 (5)
H38E	0.9751	0.3642	0.2563	0.070*	0.780 (5)
H38F	0.9972	0.2559	0.2573	0.070*	0.780 (5)
C36Y	0.9101 (15)	0.3307 (12)	0.3332 (6)	0.0334 (11)	0.220 (5)
H36E	0.8263	0.3306	0.3377	0.040*	0.220 (5)
H36F	0.9377	0.2721	0.3428	0.040*	0.220 (5)
C37Y	0.9395 (16)	0.3313 (14)	0.2715 (7)	0.0378 (12)	0.220 (5)
H37E	0.8921	0.2807	0.2470	0.045*	0.220 (5)
H37F	0.9166	0.3926	0.2638	0.045*	0.220 (5)
C38Y	1.060 (4)	0.318 (7)	0.255 (2)	0.047 (2)	0.220 (5)
H38G	1.0648	0.3064	0.2137	0.070*	0.220 (5)
H38H	1.0882	0.2635	0.2679	0.070*	0.220 (5)
H38I	1.1055	0.3751	0.2716	0.070*	0.220 (5)

### Atomic displacement parameters (Å^2^)

**Table:**

	*U*^11^	*U*^22^	*U*^33^	*U*^12^	*U*^13^	*U*^23^
Sn1A	0.01701 (12)	0.01512 (11)	0.02084 (12)	0.00071 (8)	0.00095 (9)	0.00666 (9)
Sn2A	0.01649 (11)	0.01557 (11)	0.01643 (12)	0.00109 (8)	0.00161 (9)	0.00456 (9)
O1A	0.0261 (13)	0.0214 (12)	0.0255 (13)	0.0006 (10)	0.0029 (10)	0.0102 (10)
O2A	0.0236 (12)	0.0175 (11)	0.0185 (12)	0.0011 (9)	0.0037 (9)	0.0076 (9)
O3A	0.0193 (13)	0.0192 (12)	0.0379 (15)	0.0004 (10)	0.0064 (11)	0.0018 (11)
O4A	0.0199 (12)	0.0148 (11)	0.0212 (12)	0.0032 (9)	0.0047 (9)	0.0086 (9)
O5A	0.0245 (13)	0.0189 (12)	0.0276 (13)	0.0063 (10)	0.0058 (10)	0.0080 (10)
N1A	0.0271 (16)	0.0282 (16)	0.0204 (16)	−0.0007 (12)	0.0065 (12)	0.0061 (13)
N2A	0.0212 (15)	0.0187 (14)	0.0318 (17)	0.0026 (12)	0.0059 (12)	0.0041 (12)
C1A	0.0176 (17)	0.0200 (17)	0.0194 (18)	−0.0032 (13)	−0.0040 (13)	0.0060 (14)
C2A	0.0188 (17)	0.0223 (17)	0.0170 (17)	−0.0041 (13)	−0.0031 (13)	0.0058 (14)
C3A	0.0250 (18)	0.0206 (17)	0.0214 (18)	−0.0011 (14)	−0.0001 (14)	0.0075 (14)
C4A	0.0270 (19)	0.0248 (18)	0.0193 (18)	−0.0059 (14)	−0.0001 (14)	0.0070 (14)
C5A	0.0219 (18)	0.0278 (18)	0.0163 (17)	−0.0030 (14)	0.0001 (14)	0.0049 (14)
C6A	0.029 (2)	0.0236 (18)	0.0245 (19)	0.0041 (15)	0.0040 (15)	0.0113 (15)
C7A	0.0241 (18)	0.0256 (18)	0.0179 (17)	0.0010 (14)	0.0017 (14)	0.0110 (14)
C8A	0.036 (2)	0.0240 (19)	0.025 (2)	−0.0056 (15)	0.0105 (16)	0.0043 (15)
C9A	0.052 (3)	0.032 (2)	0.024 (2)	−0.0060 (18)	0.0030 (18)	0.0108 (17)
C10A	0.032 (2)	0.030 (2)	0.026 (2)	0.0066 (16)	0.0080 (16)	0.0060 (16)
C11A	0.040 (3)	0.053 (3)	0.040 (3)	0.011 (2)	0.004 (2)	0.012 (2)
C12A	0.0202 (17)	0.0216 (17)	0.0147 (17)	0.0018 (13)	−0.0002 (13)	0.0014 (13)
C13A	0.0175 (17)	0.0208 (17)	0.0184 (17)	0.0019 (13)	−0.0005 (13)	0.0050 (13)
C14A	0.0235 (18)	0.0182 (17)	0.0217 (18)	−0.0005 (13)	0.0000 (14)	0.0063 (14)
C15A	0.0190 (17)	0.0233 (18)	0.0266 (19)	−0.0012 (14)	0.0046 (14)	0.0056 (15)
C16A	0.0166 (17)	0.0190 (16)	0.0232 (18)	0.0004 (13)	−0.0018 (13)	0.0021 (14)
C17A	0.0240 (18)	0.0152 (16)	0.029 (2)	0.0004 (13)	0.0004 (15)	0.0065 (14)
C18A	0.0180 (17)	0.0228 (17)	0.0225 (18)	−0.0013 (13)	0.0025 (14)	0.0061 (14)
C19A	0.0208 (19)	0.0233 (19)	0.046 (2)	0.0035 (14)	0.0097 (16)	0.0019 (17)
C20A	0.054 (3)	0.035 (2)	0.040 (3)	0.007 (2)	0.019 (2)	0.0045 (19)
C21A	0.0219 (18)	0.0175 (17)	0.034 (2)	0.0018 (14)	0.0003 (15)	0.0024 (15)
C22A	0.031 (2)	0.032 (2)	0.039 (2)	0.0069 (16)	−0.0017 (17)	0.0100 (18)
C23A	0.030 (2)	0.032 (2)	0.043 (2)	0.0056 (16)	−0.0080 (18)	0.0147 (18)
C24A	0.039 (2)	0.053 (3)	0.035 (2)	−0.006 (2)	−0.0027 (19)	0.014 (2)
C25A	0.045 (3)	0.053 (3)	0.039 (3)	−0.003 (2)	−0.001 (2)	0.016 (2)
C26A	0.052 (3)	0.045 (3)	0.045 (3)	−0.002 (2)	0.001 (2)	0.008 (2)
C27A	0.0178 (17)	0.0192 (17)	0.0278 (19)	−0.0013 (13)	0.0014 (14)	0.0038 (14)
C28A	0.0225 (18)	0.0193 (17)	0.0249 (19)	−0.0034 (14)	0.0045 (14)	−0.0008 (14)
C29A	0.026 (2)	0.028 (2)	0.033 (2)	−0.0049 (15)	−0.0027 (16)	0.0005 (16)
C30A	0.028 (2)	0.051 (3)	0.029 (2)	−0.0108 (18)	0.0019 (17)	−0.0003 (18)
C31A	0.039 (2)	0.0218 (18)	0.025 (2)	−0.0049 (16)	−0.0025 (16)	0.0038 (15)
C32A	0.039 (2)	0.033 (2)	0.046 (3)	0.0017 (18)	−0.0087 (19)	0.0046 (19)
C33A	0.051 (3)	0.071 (3)	0.056 (3)	−0.008 (3)	−0.019 (2)	0.022 (3)
C34A	0.055 (2)	0.0416 (17)	0.0341 (17)	0.0113 (15)	−0.0026 (14)	0.0101 (14)
C35A	0.055 (2)	0.0416 (17)	0.0341 (17)	0.0113 (15)	−0.0026 (14)	0.0101 (14)
C36A	0.0178 (17)	0.0255 (18)	0.0253 (19)	0.0006 (14)	0.0018 (14)	0.0080 (15)
C37A	0.0269 (19)	0.0243 (18)	0.0257 (19)	−0.0015 (14)	0.0002 (15)	0.0101 (15)
C38A	0.033 (2)	0.040 (2)	0.038 (2)	−0.0074 (17)	−0.0136 (18)	0.0146 (19)
Sn1B	0.02030 (12)	0.01283 (11)	0.02002 (12)	−0.00190 (8)	0.00079 (9)	0.00399 (9)
Sn2B	0.01734 (12)	0.01335 (11)	0.01678 (12)	−0.00357 (8)	−0.00010 (9)	0.00249 (9)
O1B	0.0354 (15)	0.0261 (14)	0.0429 (16)	−0.0086 (11)	−0.0141 (12)	0.0149 (12)
O2B	0.0345 (15)	0.0252 (13)	0.0361 (15)	−0.0080 (11)	−0.0155 (12)	0.0173 (12)
O3B	0.041 (3)	0.0283 (18)	0.016 (3)	−0.0181 (18)	−0.004 (2)	0.0054 (18)
O3Y	0.041 (3)	0.0283 (18)	0.016 (3)	−0.0181 (18)	−0.004 (2)	0.0054 (18)
O4B	0.0180 (11)	0.0111 (11)	0.0236 (12)	−0.0021 (9)	−0.0040 (9)	0.0036 (9)
O5B	0.0391 (16)	0.0189 (13)	0.0447 (17)	−0.0096 (11)	−0.0129 (13)	0.0100 (12)
N1B	0.0392 (19)	0.0234 (16)	0.0232 (16)	0.0052 (13)	−0.0086 (14)	0.0054 (13)
N2B	0.0273 (17)	0.0169 (15)	0.049 (2)	−0.0080 (12)	−0.0081 (14)	0.0038 (14)
C1B	0.0255 (19)	0.0247 (19)	0.031 (2)	−0.0038 (15)	−0.0062 (15)	0.0105 (16)
C2B	0.0254 (19)	0.0242 (18)	0.0234 (19)	0.0013 (14)	−0.0009 (15)	0.0096 (15)
C3B	0.030 (2)	0.0199 (17)	0.027 (2)	0.0008 (14)	0.0014 (15)	0.0092 (15)
C4B	0.035 (2)	0.0220 (18)	0.025 (2)	0.0045 (15)	0.0001 (16)	0.0114 (15)
C5B	0.030 (2)	0.0229 (18)	0.0188 (18)	0.0060 (14)	0.0016 (14)	0.0049 (14)
C6B	0.031 (2)	0.0175 (17)	0.0235 (19)	0.0027 (14)	0.0032 (15)	0.0023 (14)
C7B	0.0263 (19)	0.0246 (18)	0.0227 (19)	0.0034 (14)	0.0015 (15)	0.0112 (15)
C8B	0.048 (2)	0.027 (2)	0.029 (2)	0.0079 (17)	−0.0168 (18)	0.0049 (16)
C9B	0.067 (3)	0.039 (2)	0.030 (2)	0.012 (2)	−0.008 (2)	0.0159 (19)
C10B	0.035 (2)	0.0258 (19)	0.0228 (19)	0.0013 (16)	−0.0084 (16)	−0.0013 (15)
C11B	0.036 (2)	0.041 (2)	0.039 (2)	−0.0006 (18)	−0.0045 (19)	0.0027 (19)
C12B	0.0221 (18)	0.0172 (17)	0.0204 (18)	−0.0040 (13)	0.0046 (14)	−0.0002 (14)
C13B	0.0229 (18)	0.0153 (16)	0.0190 (17)	−0.0039 (13)	0.0037 (14)	0.0009 (13)
C14B	0.029 (2)	0.0166 (17)	0.034 (2)	−0.0039 (14)	−0.0040 (16)	0.0064 (15)
C15B	0.026 (2)	0.0213 (18)	0.040 (2)	−0.0033 (15)	−0.0124 (16)	0.0068 (16)
C16B	0.0231 (19)	0.0177 (17)	0.033 (2)	−0.0037 (14)	−0.0022 (15)	0.0015 (15)
C17B	0.030 (2)	0.0163 (17)	0.029 (2)	−0.0044 (14)	0.0017 (15)	0.0073 (15)
C18B	0.0221 (18)	0.0226 (17)	0.0216 (18)	−0.0042 (14)	−0.0025 (14)	0.0042 (14)
C19B	0.025 (2)	0.0219 (19)	0.075 (3)	−0.0035 (16)	−0.021 (2)	0.003 (2)
C20B	0.059 (3)	0.042 (3)	0.048 (3)	0.002 (2)	−0.026 (2)	−0.006 (2)
C21B	0.035 (2)	0.0178 (17)	0.034 (2)	−0.0084 (15)	0.0067 (17)	−0.0004 (15)
C22B	0.053 (3)	0.049 (3)	0.047 (3)	−0.023 (2)	0.018 (2)	−0.004 (2)
C23B	0.021 (3)	0.016 (3)	0.017 (3)	0.004 (2)	0.002 (2)	0.004 (2)
C24B	0.025 (3)	0.023 (3)	0.025 (3)	−0.001 (2)	0.000 (2)	0.003 (2)
C25B	0.024 (3)	0.029 (3)	0.021 (3)	0.009 (2)	0.004 (2)	0.005 (3)
C26B	0.027 (3)	0.030 (3)	0.023 (4)	0.008 (2)	0.001 (4)	0.006 (4)
C23Y	0.023 (7)	0.032 (6)	0.036 (7)	−0.004 (5)	−0.012 (5)	0.005 (5)
C24Y	0.023 (5)	0.026 (5)	0.037 (6)	0.001 (4)	−0.010 (4)	−0.003 (4)
C25Y	0.031 (7)	0.029 (6)	0.039 (8)	−0.004 (5)	−0.009 (7)	0.001 (7)
C26Y	0.052 (9)	0.064 (11)	0.045 (10)	0.002 (8)	0.004 (9)	−0.003 (9)
C27B	0.037 (2)	0.029 (2)	0.031 (2)	0.0039 (16)	0.0046 (17)	0.0117 (17)
C28B	0.034 (2)	0.034 (2)	0.037 (2)	0.0004 (17)	0.0074 (18)	0.0122 (18)
C29B	0.042 (3)	0.038 (2)	0.051 (3)	−0.0059 (19)	0.019 (2)	0.002 (2)
C30B	0.042 (3)	0.043 (3)	0.070 (3)	−0.001 (2)	0.022 (2)	−0.006 (2)
C31B	0.0218 (18)	0.0286 (19)	0.0220 (19)	−0.0003 (14)	0.0018 (14)	0.0025 (15)
C32B	0.026 (2)	0.031 (2)	0.030 (2)	−0.0006 (15)	0.0052 (16)	0.0070 (16)
C33B	0.025 (2)	0.033 (2)	0.036 (2)	−0.0005 (16)	−0.0004 (16)	0.0064 (17)
C34B	0.031 (2)	0.044 (3)	0.051 (3)	−0.0090 (19)	−0.0045 (19)	−0.003 (2)
C35B	0.036 (2)	0.0272 (19)	0.025 (2)	−0.0009 (16)	0.0051 (16)	0.0028 (16)
C36B	0.038 (3)	0.036 (3)	0.026 (3)	0.003 (2)	−0.001 (2)	0.006 (2)
C37B	0.044 (3)	0.038 (3)	0.030 (3)	0.011 (2)	0.014 (2)	0.003 (2)
C38B	0.059 (7)	0.041 (3)	0.044 (3)	0.006 (6)	0.022 (5)	0.016 (2)
C36Y	0.038 (3)	0.036 (3)	0.026 (3)	0.003 (2)	−0.001 (2)	0.006 (2)
C37Y	0.044 (3)	0.038 (3)	0.030 (3)	0.011 (2)	0.014 (2)	0.003 (2)
C38Y	0.059 (7)	0.041 (3)	0.044 (3)	0.006 (6)	0.022 (5)	0.016 (2)

### Geometric parameters (Å, °)

**Table:**

Sn1A—O4A	2.050 (2)	O3B—C12B	1.263 (5)
Sn1A—C27A	2.128 (3)	O3Y—C12B	1.259 (18)
Sn1A—C23A	2.150 (4)	O4B—Sn2B^ii^	2.033 (2)
Sn1A—O2A	2.183 (2)	O5B—C12B	1.257 (4)
Sn1A—O3A	2.232 (2)	O5B—Sn2B^ii^	2.210 (2)
Sn2A—O4A^i^	2.045 (2)	N1B—C5B	1.370 (4)
Sn2A—C35A	2.132 (4)	N1B—C10B	1.458 (4)
Sn2A—C31A	2.135 (3)	N1B—C8B	1.465 (4)
Sn2A—O4A	2.176 (2)	N2B—C16B	1.371 (4)
Sn2A—O5A^i^	2.255 (2)	N2B—C21B	1.462 (5)
O1A—C1A	1.248 (4)	N2B—C19B	1.469 (5)
O2A—C1A	1.304 (4)	C1B—C2B	1.480 (5)
O3A—C12A	1.266 (4)	C2B—C3B	1.389 (5)
O4A—Sn2A^i^	2.045 (2)	C2B—C7B	1.394 (5)
O5A—C12A	1.274 (4)	C3B—C4B	1.373 (5)
O5A—Sn2A^i^	2.255 (2)	C3B—H3BA	0.9500
N1A—C5A	1.370 (4)	C4B—C5B	1.410 (5)
N1A—C10A	1.464 (4)	C4B—H4BA	0.9500
N1A—C8A	1.464 (4)	C5B—C6B	1.413 (5)
N2A—C16A	1.373 (4)	C6B—C7B	1.375 (5)
N2A—C19A	1.455 (4)	C6B—H6BA	0.9500
N2A—C21A	1.460 (4)	C7B—H7BA	0.9500
C1A—C2A	1.478 (5)	C8B—C9B	1.515 (6)
C2A—C7A	1.393 (5)	C8B—H8BA	0.9900
C2A—C3A	1.405 (4)	C8B—H8BB	0.9900
C3A—C4A	1.369 (5)	C9B—H9BA	0.9800
C3A—H3AA	0.9500	C9B—H9BB	0.9800
C4A—C5A	1.412 (5)	C9B—H9BC	0.9800
C4A—H4AA	0.9500	C10B—C11B	1.527 (5)
C5A—C6A	1.415 (5)	C10B—H10C	0.9900
C6A—C7A	1.377 (5)	C10B—H10D	0.9900
C6A—H6AA	0.9500	C11B—H11D	0.9800
C7A—H7AA	0.9500	C11B—H11E	0.9800
C8A—C9A	1.518 (5)	C11B—H11F	0.9800
C8A—H8AA	0.9900	C12B—C13B	1.483 (4)
C8A—H8AB	0.9900	C13B—C14B	1.386 (5)
C9A—H9AA	0.9800	C13B—C18B	1.395 (5)
C9A—H9AB	0.9800	C14B—C15B	1.374 (5)
C9A—H9AC	0.9800	C14B—H14B	0.9500
C10A—C11A	1.517 (5)	C15B—C16B	1.414 (5)
C10A—H10A	0.9900	C15B—H15B	0.9500
C10A—H10B	0.9900	C16B—C17B	1.406 (5)
C11A—H11A	0.9800	C17B—C18B	1.375 (4)
C11A—H11B	0.9800	C17B—H17B	0.9500
C11A—H11C	0.9800	C18B—H18B	0.9500
C12A—C13A	1.479 (4)	C19B—C20B	1.509 (6)
C13A—C14A	1.395 (4)	C19B—H19C	0.9900
C13A—C18A	1.400 (4)	C19B—H19D	0.9900
C14A—C15A	1.376 (5)	C20B—H20D	0.9800
C14A—H14A	0.9500	C20B—H20E	0.9800
C15A—C16A	1.412 (5)	C20B—H20F	0.9800
C15A—H15A	0.9500	C21B—C22B	1.513 (5)
C16A—C17A	1.408 (5)	C21B—H21C	0.9900
C17A—C18A	1.378 (5)	C21B—H21D	0.9900
C17A—H17A	0.9500	C22B—H22D	0.9800
C18A—H18A	0.9500	C22B—H22E	0.9800
C19A—C20A	1.514 (6)	C22B—H22F	0.9800
C19A—H19A	0.9900	C23B—C24B	1.509 (7)
C19A—H19B	0.9900	C23B—H23B	0.9900
C20A—H20A	0.9800	C23B—H23F	0.9900
C20A—H20B	0.9800	C24B—C25B	1.545 (7)
C20A—H20C	0.9800	C24B—H24C	0.9900
C21A—C22A	1.521 (5)	C24B—H24D	0.9900
C21A—H21A	0.9900	C25B—C26B	1.530 (8)
C21A—H21B	0.9900	C25B—H25C	0.9900
C22A—H22A	0.9800	C25B—H25D	0.9900
C22A—H22B	0.9800	C26B—H26D	0.9800
C22A—H22C	0.9800	C26B—H26E	0.9800
C23A—C24A	1.488 (5)	C26B—H26F	0.9800
C23A—H23A	0.9900	C23Y—C24Y	1.514 (13)
C23A—H23E	0.9900	C23Y—H23C	0.9900
C24A—C25A	1.556 (6)	C23Y—H23D	0.9900
C24A—H24A	0.9900	C24Y—C25Y	1.539 (13)
C24A—H24B	0.9900	C24Y—H24E	0.9900
C25A—C26A	1.475 (6)	C24Y—H24F	0.9900
C25A—H25A	0.9900	C25Y—C26Y	1.529 (15)
C25A—H25B	0.9900	C25Y—H25E	0.9900
C26A—H26A	0.9800	C25Y—H25F	0.9900
C26A—H26B	0.9800	C26Y—H26G	0.9800
C26A—H26C	0.9800	C26Y—H26H	0.9800
C27A—C28A	1.526 (5)	C26Y—H26I	0.9800
C27A—H27A	0.9900	C27B—C28B	1.504 (5)
C27A—H27B	0.9900	C27B—H27C	0.9900
C28A—C29A	1.527 (4)	C27B—H27D	0.9900
C28A—H28A	0.9900	C28B—C29B	1.544 (5)
C28A—H28B	0.9900	C28B—H28C	0.9900
C29A—C30A	1.513 (5)	C28B—H28D	0.9900
C29A—H29A	0.9900	C29B—C30B	1.518 (6)
C29A—H29B	0.9900	C29B—H29C	0.9900
C30A—H30A	0.9800	C29B—H29D	0.9900
C30A—H30B	0.9800	C30B—H30D	0.9800
C30A—H30C	0.9800	C30B—H30E	0.9800
C31A—C32A	1.495 (5)	C30B—H30F	0.9800
C31A—H31A	0.9900	C31B—C32B	1.522 (5)
C31A—H31C	0.9900	C31B—H31B	0.9900
C32A—C33A	1.558 (6)	C31B—H31D	0.9900
C32A—H32A	0.9900	C32B—C33B	1.523 (5)
C32A—H32B	0.9900	C32B—H32C	0.9900
C33A—C34A	1.447 (6)	C32B—H32D	0.9900
C33A—H33A	0.9900	C33B—C34B	1.531 (5)
C33A—H33B	0.9900	C33B—H33C	0.9900
C34A—H34A	0.9800	C33B—H33D	0.9900
C34A—H34B	0.9800	C34B—H34D	0.9800
C34A—H34C	0.9800	C34B—H34E	0.9800
C35A—C36A	1.521 (5)	C34B—H34F	0.9800
C35A—H35A	0.9900	C35B—C36B	1.481 (6)
C35A—H35D	0.9900	C35B—C36Y	1.516 (17)
C36A—C37A	1.531 (4)	C35B—H35B	0.9900
C36A—H36A	0.9900	C35B—H35C	0.9900
C36A—H36B	0.9900	C35B—H35E	0.9600
C37A—C38A	1.521 (5)	C35B—H35F	0.9599
C37A—H37A	0.9900	C36B—C37B	1.538 (6)
C37A—H37B	0.9900	C36B—H35E	0.8181
C38A—H38A	0.9800	C36B—H36C	0.9900
C38A—H38B	0.9800	C36B—H36D	0.9900
C38A—H38C	0.9800	C37B—C38B	1.504 (18)
Sn1B—O4B	2.045 (2)	C37B—H37C	0.9900
Sn1B—C23Y	2.124 (13)	C37B—H37D	0.9900
Sn1B—C23B	2.135 (5)	C38B—H38D	0.9800
Sn1B—C27B	2.135 (4)	C38B—H38E	0.9800
Sn1B—O2B	2.183 (2)	C38B—H38F	0.9800
Sn1B—O3Y	2.268 (15)	C36Y—C37Y	1.542 (16)
Sn1B—O3B	2.273 (4)	C36Y—H36E	0.9900
Sn2B—O4B^ii^	2.033 (2)	C36Y—H36F	0.9900
Sn2B—C31B	2.130 (3)	C37Y—C38Y	1.48 (3)
Sn2B—C35B	2.134 (4)	C37Y—H37E	0.9900
Sn2B—O4B	2.183 (2)	C37Y—H37F	0.9900
Sn2B—O5B^ii^	2.210 (2)	C38Y—H38G	0.9800
O1B—C1B	1.245 (4)	C38Y—H38H	0.9800
O2B—C1B	1.305 (4)	C38Y—H38I	0.9800
			
O4A—Sn1A—C27A	109.30 (11)	O4B—Sn2B—O5B^ii^	167.73 (9)
O4A—Sn1A—C23A	105.41 (12)	C1B—O2B—Sn1B	100.21 (19)
C27A—Sn1A—C23A	144.73 (14)	C12B—O3B—Sn1B	133.3 (4)
O4A—Sn1A—O2A	82.88 (8)	C12B—O3Y—Sn1B	134.0 (12)
C27A—Sn1A—O2A	95.25 (11)	Sn2B^ii^—O4B—Sn1B	132.96 (10)
C23A—Sn1A—O2A	95.04 (12)	Sn2B^ii^—O4B—Sn2B	104.08 (9)
O4A—Sn1A—O3A	89.76 (8)	Sn1B—O4B—Sn2B	122.58 (10)
C27A—Sn1A—O3A	82.95 (11)	C12B—O5B—Sn2B^ii^	136.2 (2)
C23A—Sn1A—O3A	91.20 (13)	C5B—N1B—C10B	122.5 (3)
O2A—Sn1A—O3A	171.40 (8)	C5B—N1B—C8B	122.0 (3)
O4A^i^—Sn2A—C35A	116.90 (13)	C10B—N1B—C8B	115.3 (3)
O4A^i^—Sn2A—C31A	112.62 (12)	C16B—N2B—C21B	121.7 (3)
C35A—Sn2A—C31A	129.62 (15)	C16B—N2B—C19B	121.4 (3)
O4A^i^—Sn2A—O4A	75.58 (9)	C21B—N2B—C19B	116.7 (3)
C35A—Sn2A—O4A	97.68 (13)	O1B—C1B—O2B	120.1 (3)
C31A—Sn2A—O4A	102.88 (12)	O1B—C1B—C2B	121.6 (3)
O4A^i^—Sn2A—O5A^i^	92.91 (8)	O2B—C1B—C2B	118.3 (3)
C35A—Sn2A—O5A^i^	84.08 (13)	C3B—C2B—C7B	117.7 (3)
C31A—Sn2A—O5A^i^	84.82 (12)	C3B—C2B—C1B	119.4 (3)
O4A—Sn2A—O5A^i^	167.91 (8)	C7B—C2B—C1B	122.8 (3)
C1A—O2A—Sn1A	102.72 (19)	C4B—C3B—C2B	121.3 (3)
C12A—O3A—Sn1A	135.4 (2)	C4B—C3B—H3BA	119.3
Sn2A^i^—O4A—Sn1A	132.46 (11)	C2B—C3B—H3BA	119.3
Sn2A^i^—O4A—Sn2A	104.42 (9)	C3B—C4B—C5B	121.8 (3)
Sn1A—O4A—Sn2A	121.83 (10)	C3B—C4B—H4BA	119.1
C12A—O5A—Sn2A^i^	129.6 (2)	C5B—C4B—H4BA	119.1
C5A—N1A—C10A	121.9 (3)	N1B—C5B—C4B	121.7 (3)
C5A—N1A—C8A	122.5 (3)	N1B—C5B—C6B	122.0 (3)
C10A—N1A—C8A	115.5 (3)	C4B—C5B—C6B	116.2 (3)
C16A—N2A—C19A	122.0 (3)	C7B—C6B—C5B	121.3 (3)
C16A—N2A—C21A	121.8 (3)	C7B—C6B—H6BA	119.4
C19A—N2A—C21A	116.1 (3)	C5B—C6B—H6BA	119.4
O1A—C1A—O2A	120.5 (3)	C6B—C7B—C2B	121.6 (3)
O1A—C1A—C2A	121.5 (3)	C6B—C7B—H7BA	119.2
O2A—C1A—C2A	118.0 (3)	C2B—C7B—H7BA	119.2
C7A—C2A—C3A	117.2 (3)	N1B—C8B—C9B	112.0 (3)
C7A—C2A—C1A	122.6 (3)	N1B—C8B—H8BA	109.2
C3A—C2A—C1A	120.2 (3)	C9B—C8B—H8BA	109.2
C4A—C3A—C2A	121.6 (3)	N1B—C8B—H8BB	109.2
C4A—C3A—H3AA	119.2	C9B—C8B—H8BB	109.2
C2A—C3A—H3AA	119.2	H8BA—C8B—H8BB	107.9
C3A—C4A—C5A	121.4 (3)	C8B—C9B—H9BA	109.5
C3A—C4A—H4AA	119.3	C8B—C9B—H9BB	109.5
C5A—C4A—H4AA	119.3	H9BA—C9B—H9BB	109.5
N1A—C5A—C4A	121.8 (3)	C8B—C9B—H9BC	109.5
N1A—C5A—C6A	121.3 (3)	H9BA—C9B—H9BC	109.5
C4A—C5A—C6A	116.9 (3)	H9BB—C9B—H9BC	109.5
C7A—C6A—C5A	120.8 (3)	N1B—C10B—C11B	112.9 (3)
C7A—C6A—H6AA	119.6	N1B—C10B—H10C	109.0
C5A—C6A—H6AA	119.6	C11B—C10B—H10C	109.0
C6A—C7A—C2A	122.1 (3)	N1B—C10B—H10D	109.0
C6A—C7A—H7AA	119.0	C11B—C10B—H10D	109.0
C2A—C7A—H7AA	119.0	H10C—C10B—H10D	107.8
N1A—C8A—C9A	112.6 (3)	C10B—C11B—H11D	109.5
N1A—C8A—H8AA	109.1	C10B—C11B—H11E	109.5
C9A—C8A—H8AA	109.1	H11D—C11B—H11E	109.5
N1A—C8A—H8AB	109.1	C10B—C11B—H11F	109.5
C9A—C8A—H8AB	109.1	H11D—C11B—H11F	109.5
H8AA—C8A—H8AB	107.8	H11E—C11B—H11F	109.5
C8A—C9A—H9AA	109.5	O5B—C12B—O3Y	116.0 (8)
C8A—C9A—H9AB	109.5	O5B—C12B—O3B	123.7 (3)
H9AA—C9A—H9AB	109.5	O5B—C12B—C13B	118.8 (3)
C8A—C9A—H9AC	109.5	O3Y—C12B—C13B	118.5 (8)
H9AA—C9A—H9AC	109.5	O3B—C12B—C13B	117.4 (3)
H9AB—C9A—H9AC	109.5	C14B—C13B—C18B	117.7 (3)
N1A—C10A—C11A	113.1 (3)	C14B—C13B—C12B	121.4 (3)
N1A—C10A—H10A	109.0	C18B—C13B—C12B	120.9 (3)
C11A—C10A—H10A	109.0	C15B—C14B—C13B	122.0 (3)
N1A—C10A—H10B	109.0	C15B—C14B—H14B	119.0
C11A—C10A—H10B	109.0	C13B—C14B—H14B	119.0
H10A—C10A—H10B	107.8	C14B—C15B—C16B	120.7 (3)
C10A—C11A—H11A	109.5	C14B—C15B—H15B	119.6
C10A—C11A—H11B	109.5	C16B—C15B—H15B	119.6
H11A—C11A—H11B	109.5	N2B—C16B—C17B	121.8 (3)
C10A—C11A—H11C	109.5	N2B—C16B—C15B	121.4 (3)
H11A—C11A—H11C	109.5	C17B—C16B—C15B	116.8 (3)
H11B—C11A—H11C	109.5	C18B—C17B—C16B	121.5 (3)
O3A—C12A—O5A	123.4 (3)	C18B—C17B—H17B	119.2
O3A—C12A—C13A	117.3 (3)	C16B—C17B—H17B	119.2
O5A—C12A—C13A	119.4 (3)	C17B—C18B—C13B	121.2 (3)
C14A—C13A—C18A	117.6 (3)	C17B—C18B—H18B	119.4
C14A—C13A—C12A	122.0 (3)	C13B—C18B—H18B	119.4
C18A—C13A—C12A	120.4 (3)	N2B—C19B—C20B	113.1 (3)
C15A—C14A—C13A	121.4 (3)	N2B—C19B—H19C	109.0
C15A—C14A—H14A	119.3	C20B—C19B—H19C	109.0
C13A—C14A—H14A	119.3	N2B—C19B—H19D	109.0
C14A—C15A—C16A	121.4 (3)	C20B—C19B—H19D	109.0
C14A—C15A—H15A	119.3	H19C—C19B—H19D	107.8
C16A—C15A—H15A	119.3	C19B—C20B—H20D	109.5
N2A—C16A—C17A	121.3 (3)	C19B—C20B—H20E	109.5
N2A—C16A—C15A	121.9 (3)	H20D—C20B—H20E	109.5
C17A—C16A—C15A	116.8 (3)	C19B—C20B—H20F	109.5
C18A—C17A—C16A	121.3 (3)	H20D—C20B—H20F	109.5
C18A—C17A—H17A	119.3	H20E—C20B—H20F	109.5
C16A—C17A—H17A	119.3	N2B—C21B—C22B	112.2 (3)
C17A—C18A—C13A	121.3 (3)	N2B—C21B—H21C	109.2
C17A—C18A—H18A	119.3	C22B—C21B—H21C	109.2
C13A—C18A—H18A	119.3	N2B—C21B—H21D	109.2
N2A—C19A—C20A	113.4 (3)	C22B—C21B—H21D	109.2
N2A—C19A—H19A	108.9	H21C—C21B—H21D	107.9
C20A—C19A—H19A	108.9	C21B—C22B—H22D	109.5
N2A—C19A—H19B	108.9	C21B—C22B—H22E	109.5
C20A—C19A—H19B	108.9	H22D—C22B—H22E	109.5
H19A—C19A—H19B	107.7	C21B—C22B—H22F	109.5
C19A—C20A—H20A	109.5	H22D—C22B—H22F	109.5
C19A—C20A—H20B	109.5	H22E—C22B—H22F	109.5
H20A—C20A—H20B	109.5	C24B—C23B—Sn1B	117.9 (4)
C19A—C20A—H20C	109.5	C24B—C23B—H23B	107.8
H20A—C20A—H20C	109.5	Sn1B—C23B—H23B	107.8
H20B—C20A—H20C	109.5	C24B—C23B—H23F	107.8
N2A—C21A—C22A	113.0 (3)	Sn1B—C23B—H23F	107.8
N2A—C21A—H21A	109.0	H23B—C23B—H23F	107.2
C22A—C21A—H21A	109.0	C23B—C24B—C25B	111.9 (4)
N2A—C21A—H21B	109.0	C23B—C24B—H24C	109.2
C22A—C21A—H21B	109.0	C25B—C24B—H24C	109.2
H21A—C21A—H21B	107.8	C23B—C24B—H24D	109.2
C21A—C22A—H22A	109.5	C25B—C24B—H24D	109.2
C21A—C22A—H22B	109.5	H24C—C24B—H24D	107.9
H22A—C22A—H22B	109.5	C26B—C25B—C24B	112.6 (5)
C21A—C22A—H22C	109.5	C26B—C25B—H25C	109.1
H22A—C22A—H22C	109.5	C24B—C25B—H25C	109.1
H22B—C22A—H22C	109.5	C26B—C25B—H25D	109.1
C24A—C23A—Sn1A	119.3 (3)	C24B—C25B—H25D	109.1
C24A—C23A—H23A	107.5	H25C—C25B—H25D	107.8
Sn1A—C23A—H23A	107.5	C24Y—C23Y—Sn1B	115.6 (9)
C24A—C23A—H23E	107.5	C24Y—C23Y—H23C	108.4
Sn1A—C23A—H23E	107.5	Sn1B—C23Y—H23C	108.4
H23A—C23A—H23E	107.0	C24Y—C23Y—H23D	108.4
C23A—C24A—C25A	115.7 (4)	Sn1B—C23Y—H23D	108.4
C23A—C24A—H24A	108.3	H23C—C23Y—H23D	107.4
C25A—C24A—H24A	108.3	C23Y—C24Y—C25Y	113.4 (10)
C23A—C24A—H24B	108.3	C23Y—C24Y—H24E	108.9
C25A—C24A—H24B	108.3	C25Y—C24Y—H24E	108.9
H24A—C24A—H24B	107.4	C23Y—C24Y—H24F	108.9
C26A—C25A—C24A	113.2 (4)	C25Y—C24Y—H24F	108.9
C26A—C25A—H25A	108.9	H24E—C24Y—H24F	107.7
C24A—C25A—H25A	108.9	C26Y—C25Y—C24Y	113.0 (13)
C26A—C25A—H25B	108.9	C26Y—C25Y—H25E	109.0
C24A—C25A—H25B	108.9	C24Y—C25Y—H25E	109.0
H25A—C25A—H25B	107.8	C26Y—C25Y—H25F	109.0
C25A—C26A—H26A	109.5	C24Y—C25Y—H25F	109.0
C25A—C26A—H26B	109.5	H25E—C25Y—H25F	107.8
H26A—C26A—H26B	109.5	C25Y—C26Y—H26G	109.5
C25A—C26A—H26C	109.5	C25Y—C26Y—H26H	109.5
H26A—C26A—H26C	109.5	H26G—C26Y—H26H	109.5
H26B—C26A—H26C	109.5	C25Y—C26Y—H26I	109.5
C28A—C27A—Sn1A	115.5 (2)	H26G—C26Y—H26I	109.5
C28A—C27A—H27A	108.4	H26H—C26Y—H26I	109.5
Sn1A—C27A—H27A	108.4	C28B—C27B—Sn1B	114.0 (3)
C28A—C27A—H27B	108.4	C28B—C27B—H27C	108.8
Sn1A—C27A—H27B	108.4	Sn1B—C27B—H27C	108.8
H27A—C27A—H27B	107.5	C28B—C27B—H27D	108.8
C27A—C28A—C29A	113.9 (3)	Sn1B—C27B—H27D	108.8
C27A—C28A—H28A	108.8	H27C—C27B—H27D	107.6
C29A—C28A—H28A	108.8	C27B—C28B—C29B	113.7 (3)
C27A—C28A—H28B	108.8	C27B—C28B—H28C	108.8
C29A—C28A—H28B	108.8	C29B—C28B—H28C	108.8
H28A—C28A—H28B	107.7	C27B—C28B—H28D	108.8
C30A—C29A—C28A	112.9 (3)	C29B—C28B—H28D	108.8
C30A—C29A—H29A	109.0	H28C—C28B—H28D	107.7
C28A—C29A—H29A	109.0	C30B—C29B—C28B	110.5 (4)
C30A—C29A—H29B	109.0	C30B—C29B—H29C	109.6
C28A—C29A—H29B	109.0	C28B—C29B—H29C	109.6
H29A—C29A—H29B	107.8	C30B—C29B—H29D	109.6
C29A—C30A—H30A	109.5	C28B—C29B—H29D	109.6
C29A—C30A—H30B	109.5	H29C—C29B—H29D	108.1
H30A—C30A—H30B	109.5	C29B—C30B—H30D	109.5
C29A—C30A—H30C	109.5	C29B—C30B—H30E	109.5
H30A—C30A—H30C	109.5	H30D—C30B—H30E	109.5
H30B—C30A—H30C	109.5	C29B—C30B—H30F	109.5
C32A—C31A—Sn2A	119.9 (3)	H30D—C30B—H30F	109.5
C32A—C31A—H31A	107.3	H30E—C30B—H30F	109.5
Sn2A—C31A—H31A	107.3	C32B—C31B—Sn2B	117.6 (2)
C32A—C31A—H31C	107.3	C32B—C31B—H31B	107.9
Sn2A—C31A—H31C	107.3	Sn2B—C31B—H31B	107.9
H31A—C31A—H31C	106.9	C32B—C31B—H31D	107.9
C31A—C32A—C33A	113.5 (3)	Sn2B—C31B—H31D	107.9
C31A—C32A—H32A	108.9	H31B—C31B—H31D	107.2
C33A—C32A—H32A	108.9	C31B—C32B—C33B	112.4 (3)
C31A—C32A—H32B	108.9	C31B—C32B—H32C	109.1
C33A—C32A—H32B	108.9	C33B—C32B—H32C	109.1
H32A—C32A—H32B	107.7	C31B—C32B—H32D	109.1
C34A—C33A—C32A	118.4 (4)	C33B—C32B—H32D	109.1
C34A—C33A—H33A	107.7	H32C—C32B—H32D	107.9
C32A—C33A—H33A	107.7	C32B—C33B—C34B	113.6 (3)
C34A—C33A—H33B	107.7	C32B—C33B—H33C	108.8
C32A—C33A—H33B	107.7	C34B—C33B—H33C	108.8
H33A—C33A—H33B	107.1	C32B—C33B—H33D	108.8
C33A—C34A—H34A	109.5	C34B—C33B—H33D	108.8
C33A—C34A—H34B	109.5	H33C—C33B—H33D	107.7
H34A—C34A—H34B	109.5	C33B—C34B—H34D	109.5
C33A—C34A—H34C	109.5	C33B—C34B—H34E	109.5
H34A—C34A—H34C	109.5	H34D—C34B—H34E	109.5
H34B—C34A—H34C	109.5	C33B—C34B—H34F	109.5
C36A—C35A—Sn2A	117.3 (3)	H34D—C34B—H34F	109.5
C36A—C35A—H35A	108.0	H34E—C34B—H34F	109.5
Sn2A—C35A—H35A	108.0	C36B—C35B—C36Y	78.9 (7)
C36A—C35A—H35D	108.0	C36B—C35B—Sn2B	116.8 (3)
Sn2A—C35A—H35D	108.0	C36Y—C35B—Sn2B	115.1 (6)
H35A—C35A—H35D	107.2	C36B—C35B—H35B	108.1
C35A—C36A—C37A	112.8 (3)	Sn2B—C35B—H35B	108.1
C35A—C36A—H36A	109.0	C36B—C35B—H35C	108.1
C37A—C36A—H36A	109.0	C36Y—C35B—H35C	126.8
C35A—C36A—H36B	109.0	Sn2B—C35B—H35C	108.1
C37A—C36A—H36B	109.0	H35B—C35B—H35C	107.3
H36A—C36A—H36B	107.8	C36Y—C35B—H35E	108.8
C38A—C37A—C36A	112.6 (3)	Sn2B—C35B—H35E	108.2
C38A—C37A—H37A	109.1	H35B—C35B—H35E	135.7
C36A—C37A—H37A	109.1	H35C—C35B—H35E	84.5
C38A—C37A—H37B	109.1	C36B—C35B—H35F	125.5
C36A—C37A—H37B	109.1	C36Y—C35B—H35F	109.3
H37A—C37A—H37B	107.8	Sn2B—C35B—H35F	107.9
C37A—C38A—H38A	109.5	H35B—C35B—H35F	84.6
C37A—C38A—H38B	109.5	H35E—C35B—H35F	107.3
H38A—C38A—H38B	109.5	C35B—C36B—C37B	114.2 (4)
C37A—C38A—H38C	109.5	C37B—C36B—H35E	110.1
H38A—C38A—H38C	109.5	C35B—C36B—H36C	108.7
H38B—C38A—H38C	109.5	C37B—C36B—H36C	108.7
O4B—Sn1B—C23Y	108.0 (4)	H35E—C36B—H36C	137.1
O4B—Sn1B—C23B	104.63 (17)	C35B—C36B—H36D	108.7
C23Y—Sn1B—C23B	18.2 (3)	C37B—C36B—H36D	108.7
O4B—Sn1B—C27B	107.81 (12)	H35E—C36B—H36D	76.4
C23Y—Sn1B—C27B	144.2 (4)	H36C—C36B—H36D	107.6
C23B—Sn1B—C27B	142.93 (18)	C38B—C37B—C36B	114.4 (5)
O4B—Sn1B—O2B	84.40 (8)	C38B—C37B—H37C	108.7
C23Y—Sn1B—O2B	88.7 (4)	C36B—C37B—H37C	108.7
C23B—Sn1B—O2B	106.29 (19)	C38B—C37B—H37D	108.7
C27B—Sn1B—O2B	94.36 (13)	C36B—C37B—H37D	108.7
O4B—Sn1B—O3Y	91.7 (4)	H37C—C37B—H37D	107.6
C23Y—Sn1B—O3Y	83.6 (7)	C35B—C36Y—C37Y	111.5 (13)
C23B—Sn1B—O3Y	65.6 (6)	C35B—C36Y—H36E	109.3
C27B—Sn1B—O3Y	95.8 (6)	C37Y—C36Y—H36E	109.3
O2B—Sn1B—O3Y	169.8 (6)	C35B—C36Y—H36F	109.3
O4B—Sn1B—O3B	92.07 (11)	C37Y—C36Y—H36F	109.3
C23Y—Sn1B—O3B	99.0 (4)	H36E—C36Y—H36F	108.0
C23B—Sn1B—O3B	81.3 (3)	C38Y—C37Y—C36Y	117 (2)
C27B—Sn1B—O3B	80.1 (2)	C38Y—C37Y—H37E	108.0
O2B—Sn1B—O3B	172.2 (2)	C36Y—C37Y—H37E	108.0
O3Y—Sn1B—O3B	16.4 (5)	C38Y—C37Y—H37F	108.0
O4B^ii^—Sn2B—C31B	108.92 (11)	C36Y—C37Y—H37F	108.0
O4B^ii^—Sn2B—C35B	113.40 (12)	H37E—C37Y—H37F	107.2
C31B—Sn2B—C35B	137.68 (14)	C37Y—C38Y—H38G	109.5
O4B^ii^—Sn2B—O4B	75.92 (9)	C37Y—C38Y—H38H	109.5
C31B—Sn2B—O4B	93.87 (11)	H38G—C38Y—H38H	109.5
C35B—Sn2B—O4B	95.57 (11)	C37Y—C38Y—H38I	109.5
O4B^ii^—Sn2B—O5B^ii^	92.38 (9)	H38G—C38Y—H38I	109.5
C31B—Sn2B—O5B^ii^	93.42 (12)	H38H—C38Y—H38I	109.5
C35B—Sn2B—O5B^ii^	85.62 (12)		
			
O4A—Sn1A—O2A—C1A	−179.54 (19)	O3B—Sn1B—O4B—Sn2B^ii^	5.2 (2)
C27A—Sn1A—O2A—C1A	71.6 (2)	C23Y—Sn1B—O4B—Sn2B	93.3 (4)
C23A—Sn1A—O2A—C1A	−74.6 (2)	C23B—Sn1B—O4B—Sn2B	111.9 (2)
O4A—Sn1A—O3A—C12A	40.3 (3)	C27B—Sn1B—O4B—Sn2B	−86.28 (16)
C27A—Sn1A—O3A—C12A	149.8 (3)	O2B—Sn1B—O4B—Sn2B	6.51 (12)
C23A—Sn1A—O3A—C12A	−65.1 (3)	O3Y—Sn1B—O4B—Sn2B	177.1 (6)
C27A—Sn1A—O4A—Sn2A^i^	−86.67 (17)	O3B—Sn1B—O4B—Sn2B	−166.5 (2)
C23A—Sn1A—O4A—Sn2A^i^	86.95 (18)	O4B^ii^—Sn2B—O4B—Sn2B^ii^	−0.003 (1)
O2A—Sn1A—O4A—Sn2A^i^	−179.76 (16)	C31B—Sn2B—O4B—Sn2B^ii^	108.54 (12)
O3A—Sn1A—O4A—Sn2A^i^	−4.23 (15)	C35B—Sn2B—O4B—Sn2B^ii^	−112.77 (13)
C27A—Sn1A—O4A—Sn2A	78.21 (15)	O5B^ii^—Sn2B—O4B—Sn2B^ii^	−17.8 (5)
C23A—Sn1A—O4A—Sn2A	−108.18 (15)	O4B^ii^—Sn2B—O4B—Sn1B	173.78 (19)
O2A—Sn1A—O4A—Sn2A	−14.89 (11)	C31B—Sn2B—O4B—Sn1B	−77.68 (15)
O3A—Sn1A—O4A—Sn2A	160.65 (12)	C35B—Sn2B—O4B—Sn1B	61.01 (15)
O4A^i^—Sn2A—O4A—Sn2A^i^	0.0	O5B^ii^—Sn2B—O4B—Sn1B	156.0 (4)
C35A—Sn2A—O4A—Sn2A^i^	115.89 (14)	Sn1B—O2B—C1B—O1B	−10.7 (4)
C31A—Sn2A—O4A—Sn2A^i^	−110.40 (12)	Sn1B—O2B—C1B—C2B	167.1 (3)
O5A^i^—Sn2A—O4A—Sn2A^i^	18.3 (4)	O1B—C1B—C2B—C3B	0.0 (5)
O4A^i^—Sn2A—O4A—Sn1A	−168.54 (18)	O2B—C1B—C2B—C3B	−177.8 (3)
C35A—Sn2A—O4A—Sn1A	−52.64 (16)	O1B—C1B—C2B—C7B	178.3 (3)
C31A—Sn2A—O4A—Sn1A	81.06 (15)	O2B—C1B—C2B—C7B	0.5 (5)
O5A^i^—Sn2A—O4A—Sn1A	−150.3 (3)	C7B—C2B—C3B—C4B	1.7 (5)
Sn1A—O2A—C1A—O1A	2.2 (3)	C1B—C2B—C3B—C4B	−179.9 (3)
Sn1A—O2A—C1A—C2A	−179.2 (2)	C2B—C3B—C4B—C5B	1.5 (6)
O1A—C1A—C2A—C7A	−175.2 (3)	C10B—N1B—C5B—C4B	−177.6 (3)
O2A—C1A—C2A—C7A	6.1 (5)	C8B—N1B—C5B—C4B	−2.4 (5)
O1A—C1A—C2A—C3A	5.1 (5)	C10B—N1B—C5B—C6B	2.2 (5)
O2A—C1A—C2A—C3A	−173.5 (3)	C8B—N1B—C5B—C6B	177.5 (3)
C7A—C2A—C3A—C4A	−0.2 (5)	C3B—C4B—C5B—N1B	176.5 (3)
C1A—C2A—C3A—C4A	179.4 (3)	C3B—C4B—C5B—C6B	−3.4 (5)
C2A—C3A—C4A—C5A	0.8 (5)	N1B—C5B—C6B—C7B	−177.9 (3)
C10A—N1A—C5A—C4A	175.9 (3)	C4B—C5B—C6B—C7B	2.0 (5)
C8A—N1A—C5A—C4A	−1.5 (5)	C5B—C6B—C7B—C2B	1.2 (5)
C10A—N1A—C5A—C6A	−4.8 (5)	C3B—C2B—C7B—C6B	−3.1 (5)
C8A—N1A—C5A—C6A	177.8 (3)	C1B—C2B—C7B—C6B	178.6 (3)
C3A—C4A—C5A—N1A	178.8 (3)	C5B—N1B—C8B—C9B	90.5 (4)
C3A—C4A—C5A—C6A	−0.5 (5)	C10B—N1B—C8B—C9B	−94.0 (4)
N1A—C5A—C6A—C7A	−179.5 (3)	C5B—N1B—C10B—C11B	85.4 (4)
C4A—C5A—C6A—C7A	−0.2 (5)	C8B—N1B—C10B—C11B	−90.1 (4)
C5A—C6A—C7A—C2A	0.7 (5)	Sn2B^ii^—O5B—C12B—O3Y	49.9 (12)
C3A—C2A—C7A—C6A	−0.5 (5)	Sn2B^ii^—O5B—C12B—O3B	16.7 (7)
C1A—C2A—C7A—C6A	179.8 (3)	Sn2B^ii^—O5B—C12B—C13B	−159.0 (2)
C5A—N1A—C8A—C9A	88.8 (4)	Sn1B—O3Y—C12B—O5B	−49 (2)
C10A—N1A—C8A—C9A	−88.7 (4)	Sn1B—O3Y—C12B—O3B	63.6 (19)
C5A—N1A—C10A—C11A	87.6 (4)	Sn1B—O3Y—C12B—C13B	159.5 (12)
C8A—N1A—C10A—C11A	−94.9 (4)	Sn1B—O3B—C12B—O5B	22.0 (9)
Sn1A—O3A—C12A—O5A	−31.8 (5)	Sn1B—O3B—C12B—O3Y	−62.0 (16)
Sn1A—O3A—C12A—C13A	149.0 (2)	Sn1B—O3B—C12B—C13B	−162.3 (4)
Sn2A^i^—O5A—C12A—O3A	−18.1 (5)	O5B—C12B—C13B—C14B	10.1 (5)
Sn2A^i^—O5A—C12A—C13A	161.1 (2)	O3Y—C12B—C13B—C14B	160.4 (12)
O3A—C12A—C13A—C14A	167.5 (3)	O3B—C12B—C13B—C14B	−165.8 (5)
O5A—C12A—C13A—C14A	−11.8 (5)	O5B—C12B—C13B—C18B	−171.0 (3)
O3A—C12A—C13A—C18A	−11.3 (5)	O3Y—C12B—C13B—C18B	−20.6 (13)
O5A—C12A—C13A—C18A	169.5 (3)	O3B—C12B—C13B—C18B	13.1 (6)
C18A—C13A—C14A—C15A	2.8 (5)	C18B—C13B—C14B—C15B	0.1 (5)
C12A—C13A—C14A—C15A	−176.0 (3)	C12B—C13B—C14B—C15B	179.1 (3)
C13A—C14A—C15A—C16A	−1.3 (5)	C13B—C14B—C15B—C16B	0.4 (6)
C19A—N2A—C16A—C17A	−176.0 (3)	C21B—N2B—C16B—C17B	7.3 (5)
C21A—N2A—C16A—C17A	8.8 (5)	C19B—N2B—C16B—C17B	−178.7 (4)
C19A—N2A—C16A—C15A	3.9 (5)	C21B—N2B—C16B—C15B	−172.2 (3)
C21A—N2A—C16A—C15A	−171.3 (3)	C19B—N2B—C16B—C15B	1.7 (6)
C14A—C15A—C16A—N2A	178.6 (3)	C14B—C15B—C16B—N2B	179.3 (4)
C14A—C15A—C16A—C17A	−1.4 (5)	C14B—C15B—C16B—C17B	−0.2 (5)
N2A—C16A—C17A—C18A	−177.4 (3)	N2B—C16B—C17B—C18B	−179.9 (3)
C15A—C16A—C17A—C18A	2.7 (5)	C15B—C16B—C17B—C18B	−0.4 (5)
C16A—C17A—C18A—C13A	−1.3 (5)	C16B—C17B—C18B—C13B	0.9 (5)
C14A—C13A—C18A—C17A	−1.5 (5)	C14B—C13B—C18B—C17B	−0.7 (5)
C12A—C13A—C18A—C17A	177.3 (3)	C12B—C13B—C18B—C17B	−179.7 (3)
C16A—N2A—C19A—C20A	−87.3 (4)	C16B—N2B—C19B—C20B	−82.5 (4)
C21A—N2A—C19A—C20A	88.2 (4)	C21B—N2B—C19B—C20B	91.8 (4)
C16A—N2A—C21A—C22A	−89.4 (4)	C16B—N2B—C21B—C22B	−89.5 (4)
C19A—N2A—C21A—C22A	95.1 (4)	C19B—N2B—C21B—C22B	96.3 (4)
O4A—Sn1A—C23A—C24A	−66.3 (3)	O4B—Sn1B—C23B—C24B	−79.9 (4)
C27A—Sn1A—C23A—C24A	103.3 (4)	C23Y—Sn1B—C23B—C24B	23.3 (13)
O2A—Sn1A—C23A—C24A	−150.3 (3)	C27B—Sn1B—C23B—C24B	129.7 (4)
O3A—Sn1A—C23A—C24A	23.8 (3)	O2B—Sn1B—C23B—C24B	8.5 (5)
Sn1A—C23A—C24A—C25A	156.7 (3)	O3Y—Sn1B—C23B—C24B	−165.0 (7)
C23A—C24A—C25A—C26A	55.0 (5)	O3B—Sn1B—C23B—C24B	−169.8 (5)
O4A—Sn1A—C27A—C28A	29.5 (3)	Sn1B—C23B—C24B—C25B	−178.5 (4)
C23A—Sn1A—C27A—C28A	−139.8 (3)	C23B—C24B—C25B—C26B	−68.7 (7)
O2A—Sn1A—C27A—C28A	113.8 (2)	O4B—Sn1B—C23Y—C24Y	33.1 (10)
O3A—Sn1A—C27A—C28A	−57.7 (2)	C23B—Sn1B—C23Y—C24Y	−49.0 (11)
Sn1A—C27A—C28A—C29A	−178.2 (2)	C27B—Sn1B—C23Y—C24Y	−147.6 (6)
C27A—C28A—C29A—C30A	174.6 (3)	O2B—Sn1B—C23Y—C24Y	116.8 (9)
O4A^i^—Sn2A—C31A—C32A	−86.5 (3)	O3Y—Sn1B—C23Y—C24Y	−56.6 (10)
C35A—Sn2A—C31A—C32A	104.6 (3)	O3B—Sn1B—C23Y—C24Y	−62.0 (9)
O4A—Sn2A—C31A—C32A	−7.0 (3)	Sn1B—C23Y—C24Y—C25Y	176.5 (8)
O5A^i^—Sn2A—C31A—C32A	−177.5 (3)	C23Y—C24Y—C25Y—C26Y	176.8 (13)
Sn2A—C31A—C32A—C33A	−176.9 (3)	O4B—Sn1B—C27B—C28B	−28.8 (3)
C31A—C32A—C33A—C34A	59.3 (6)	C23Y—Sn1B—C27B—C28B	152.0 (6)
O4A^i^—Sn2A—C35A—C36A	−27.8 (3)	C23B—Sn1B—C27B—C28B	121.1 (4)
C31A—Sn2A—C35A—C36A	140.7 (3)	O2B—Sn1B—C27B—C28B	−114.3 (3)
O4A—Sn2A—C35A—C36A	−105.5 (3)	O3Y—Sn1B—C27B—C28B	64.9 (5)
O5A^i^—Sn2A—C35A—C36A	62.5 (3)	O3B—Sn1B—C27B—C28B	60.2 (3)
Sn2A—C35A—C36A—C37A	175.8 (2)	Sn1B—C27B—C28B—C29B	−174.7 (3)
C35A—C36A—C37A—C38A	179.0 (3)	C27B—C28B—C29B—C30B	179.0 (3)
O4B—Sn1B—O2B—C1B	−175.6 (2)	O4B^ii^—Sn2B—C31B—C32B	−113.8 (3)
C23Y—Sn1B—O2B—C1B	76.2 (4)	C35B—Sn2B—C31B—C32B	67.2 (3)
C23B—Sn1B—O2B—C1B	80.8 (3)	O4B—Sn2B—C31B—C32B	169.8 (3)
C27B—Sn1B—O2B—C1B	−68.1 (2)	O5B^ii^—Sn2B—C31B—C32B	−20.1 (3)
O3Y—Sn1B—O2B—C1B	116 (3)	Sn2B—C31B—C32B—C33B	−68.2 (4)
O4B—Sn1B—O3B—C12B	−29.5 (7)	C31B—C32B—C33B—C34B	−174.2 (3)
C23Y—Sn1B—O3B—C12B	79.0 (7)	O4B^ii^—Sn2B—C35B—C36B	22.0 (3)
C23B—Sn1B—O3B—C12B	74.9 (7)	C31B—Sn2B—C35B—C36B	−158.9 (3)
C27B—Sn1B—O3B—C12B	−137.3 (7)	O4B—Sn2B—C35B—C36B	99.1 (3)
O3Y—Sn1B—O3B—C12B	59.5 (17)	O5B^ii^—Sn2B—C35B—C36B	−68.7 (3)
O4B—Sn1B—O3Y—C12B	30.4 (19)	O4B^ii^—Sn2B—C35B—C36Y	111.9 (8)
C23Y—Sn1B—O3Y—C12B	138 (2)	C31B—Sn2B—C35B—C36Y	−69.1 (8)
C23B—Sn1B—O3Y—C12B	136 (2)	O4B—Sn2B—C35B—C36Y	−171.1 (8)
C27B—Sn1B—O3Y—C12B	−77.7 (19)	O5B^ii^—Sn2B—C35B—C36Y	21.2 (8)
O2B—Sn1B—O3Y—C12B	98 (4)	C36Y—C35B—C36B—C37B	75.4 (7)
O3B—Sn1B—O3Y—C12B	−61 (2)	Sn2B—C35B—C36B—C37B	−172.0 (3)
C23Y—Sn1B—O4B—Sn2B^ii^	−95.0 (4)	C35B—C36B—C37B—C38B	−57.3 (11)
C23B—Sn1B—O4B—Sn2B^ii^	−76.3 (2)	C36B—C35B—C36Y—C37Y	−79.5 (13)
C27B—Sn1B—O4B—Sn2B^ii^	85.47 (18)	Sn2B—C35B—C36Y—C37Y	166.0 (10)
O2B—Sn1B—O4B—Sn2B^ii^	178.25 (16)	C35B—C36Y—C37Y—C38Y	67 (5)
O3Y—Sn1B—O4B—Sn2B^ii^	−11.2 (6)		

Symmetry codes: (i) −*x*+1, −*y*+2, −*z*; (ii) −*x*+2, −*y*+1, −*z*+1.

### Hydrogen-bond geometry (Å, °)

**Table:**

*D*—H···*A*	*D*—H	H···*A*	*D*···*A*	*D*—H···*A*
C28A—H28A···O3A	0.99	2.56	3.140 (4)	117
C28B—H28D···O3B	0.99	2.43	3.065 (8)	121
C32A—H32B···O2A	0.99	2.47	3.252 (5)	136
